# Potential diagnostic and prognostic value and regulatory relationship of long noncoding RNA CCAT1 and miR-130a-3p in clear cell renal cell carcinoma

**DOI:** 10.1186/s12935-021-01757-7

**Published:** 2021-01-22

**Authors:** Jingjing Jing, Xu Zhao, Jiannan Wang, Tan Li

**Affiliations:** 1 Tumor Etiology and Screening Department of Cancer Institute and General Surgery, Key Laboratory of Cancer Etiology and Prevention in Liaoning Education Department, the First Hospital of China Medical University, Shenyang, Liaoning 110001 P.R. China; 2 Mathematical Computer Teaching and Research Office, Liaoning Vocational College of Medicine, Shenyang, Liaoning 110101 P.R. China; 3 Department of Ultrasound, the First Hospital of China Medical University, Shenyang, Liaoning 110001 P.R. China; 4 Department of Cardiovascular Ultrasound, the First Hospital of China Medical University, No.155 Nanjing Bei Street, Heping District, Shenyang, Liaoning 110001 P.R. China

**Keywords:** miR-130a-3p, CCAT1, ccRCC, Expression, Biological effect

## Abstract

**Background:**

MicroRNAs (miRNAs) and long non-coding RNAs (lncRNAs) could interact with each other to play a vital role in the pathogenesis of cancers. We aimed to examine the expression profile, clinical significance and regulatory relationship of miR-130a-3p and its predicted interactive lncRNA in clear cell renal cell carcinoma (ccRCC).

**Methods:**

Bioinformatics analysis was used to predict lncRNAs binding with miR-130a-3p. qRT-PCR was employed to detect the expression levels of miR-130a-3p and the miRNA-targeted lncRNA, and their clinical values in ccRCC were clarified. The lncRNA sponge potential of miR-130a-3p was assessed through dual-luciferase reporter assay and the biological effects of them were observed.

**Results:**

Colon cancer associated transcript 1 (CCAT1) directly interacted with miR-130a-3p and negatively regulated miR-130a-3p expression. CCAT1 was upregulated and miR-130a-3p was downregulated in ccRCC cell line and tissues (all *P* < 0.05). High CCAT1 and low miR-130a-3p expression was correlated with larger tumor size and advanced TNM stage in ccRCC patients. High CCAT1 level suggested a poor survival prognosis. There was a negative association between CCAT1 and miR-130a-3p expression (r = − 0.373, *P* = 0.010). MiR-130a-3p mimic and si-CCAT1 inhibited ccRCC cell proliferation and invasion, and induced apoptosis.

**Conclusions:**

CCAT1/miR-130a-3p axis may have potential to serve as a novel diagnostic and prognostic target of ccRCC patients.

## Introduction

As the most common and lethal kidney malignancy, clear cell renal cell carcinoma (ccRCC) has an increasing incidence and poor prognosis [1]. Thus far, the pathological mechanism of ccRCC remains unclear. For localized renal carcinoma, nephrectomy is the only option of curative treatment [2]. No effective preventive adjuvant therapy is available for ccRCC patients after surgical resection, and high grade ccRCC patients have a high likelihood of relapse or metastases [3]. It has been commonly accepted that early diagnosis and treatment can greatly increase the survival rate of cancer patients. Hence, it is very important to identify effective biomarkers for early detection of tumors. Uncontrolled cellular proliferation and invasion is a hallmark of all malignancies. In this regard, recognition of new molecular biomarkers will help us to improve the understanding of ccRCC and enhance the treatment and prognosis of patients with ccRCC [4].

Non-protein coding genes, also named noncoding RNAs (ncRNAs), account for more than 95% of human genome. In the ncRNAs family, the biological and clinical roles of long non-coding RNAs (lncRNAs) and microRNAs (miRNAs) have attracted wide attention. They can interact to enhance the post-transcriptional regulation [5, 6]. Recent evidence showed that ncRNAs had an important function in the pathogenesis of cancer and provided a new perspective for the biological study of the disease [7, 8]. Mounting studies also confirmed that ncRNAs played a critical role in the development of cancers, including ccRCC, through a variety of mechanisms functioning as regulatory factors. The dysregulation of miRNA was involved in mediating cell biological processes, which have been linked with the initiation and progression of ccRCC [9]. LncRNAs may serve as competing endogenous RNAs (ceRNAs) by binding to miRNAs through their miRNA response elements to competitively inhibit miRNAs activity in ccRCC [10].

MiR-130a-3p is a vertebrate-specific miRNA. A number of published researches have explored the regulatory function of miR-130a-3p in cancer cells. The expression level of miR-130a-3p was demonstrated to be decreased in ovarian cancer cells [11], prostate cancer cells [12], esophageal cancer cells [13] and hepatocellular carcinoma cells [14]. On the contrary, up-regulated expression of miR-130a-3p was observed in human cervical cancer [15]. In a recent study, Li et al. found that miR-130a-3p was down-regulated in renal carcinoma cells. Up-regulation of miR-130a-3p could inhibit the renal carcinoma cell proliferation and migration, and promote apoptosis, suggesting the tumor-suppressing effect of miR-130a-3p in renal carcinoma [16]. However, the role of miR-130a-3p in ccRCC remains unclear, especially its signaling pathway, crosstalk with some lncRNAs in the tumorigenesis. Whether predicted competitive lncRNAs regulate miR-130a-3p expression and further participate in the development and prognosis of ccRCC is unknown and deserves further investigation.

In the current study, we first investigated lncRNA that potentially served as a ceRNA of miR-130a-3p using bioinformatics methods. *In vivo*, we detected the expression of interactive lncRNA and miR-130a-3p in ccRCC tissues, and analyzed their associations with the clinicopathological parameters of ccRCC patients. The prognostic values of these two indicators were evaluated by bioinformatics analysis. *In vitro*, molecular biological experiments were applied to verify their direct binding relationship and the biological effects. Our findings will be helpful to identify possible diagnostic and prognostic markers of ccRCC.

## Materials and methods

### Clinical tissue samples

Forty-seven cases of surgically resected ccRCC tissues and the paired adjacent paracancer tissues were collected from patients undergoing radical nephrectomy at the First Hospital of China Medical University. The diagnoses were all based on histopathological evidence. The included patients did not receive radiotherapy, chemotherapy, immunotherapy, targeted therapy, interventional embolization and other adjuvant treatment before surgery, and had no history of other malignant tumors. The clinicopathological characteristics of these patients were collected from medical records. After renal operation, the tissue specimens were frozen immediately at − 80^°^C until subsequent use. This study was approved by the Ethics Committee of the First Hospital of China Medical University (Shenyang, China), and the written informed consent of each subject was obtained.

### Cell culture and transient transfection

Human renal clear cell carcinoma 786-O cell line was purchased from the Cell Bank of typical Culture Preservation Committee of Chinese Academy of Science. Renal proximal tubular epithelial cell HK-2 was purchased from Saiqi Bioengineering Co., Ltd (Shanghai, China). 293T cell line is a highly transfectable derivative of human embryonic kidney 293 cells. It was purchased from Zhongqiaoxinzhou Biotech Co., Ltd (Shanghai, China). All cell lines have been authenticated by STR analysis when purchase. 786-O cells, HK-2 cells and 293T cells were separately cultured in RPMI 1640 medium, DMEM/F12 medium and DMEM medium (Hyclone, Logan, UT, USA) with 10% fetal bovine serum (Hyclone, Logan, UT, USA) in a 5% CO_2_ atmosphere at 37 °C in an incubator (Thermo Fisher Scientific, Waltham, MA, USA). 100 pmol MiR-130a-3p mimic, small interfering RNA (si)-CCAT1, and their negative controls (NCs) were transfected into 786-O cells following the manufacturer’s protocols using Lipofectamine® 2000 (Thermo Fisher Scientific, Waltham, MA, USA). Subsequent experiments were performed following 48 h transfection.

### RNA extraction and quantitative real-time PCR (qRT-PCR)

Using Trizol buffer (Thermo Fisher Scientific, MA, USA), total RNA was extracted and then reverse transcribed to cDNA with PrimeScript RT Master Mix (Takara). Quantitative real-time PCR was performed using SYBR Premix Ex Taq (Takara, Liaoning, China) on Eppendorf equipment following the manufacturer’s instructions. U6 and β-actin were adopted as the internal controls. The 2^− ΔΔCT^ methods helped normalize the relative gene expression to β-actin or U6. The primer sequences for miR-130a-3p were: Forward, 5’-CGCCGCAGTGCAATGTTAAA-3’; reverse, 5’-GTGCAGGGTCCGAGGTATTC-3’. The sequences of the primer for the lncRNA CCAT1 were: Forward, 5’-TATCACCTACGCATACCTCT-3’; reverse, 5’-GAATTTCGTGTTCGTTTACT-3’. The sequences of the primers for U6 were: Forward, 5’-GCTTCGGCAGCACATATACT-3’; reverse, 5’-GTGCAGGGTCCGAGGTATTC-3’. The sequences of the primers for β-actin were: Forward, 5’-CACTGTGCCCATCTACGAGG-3’; reverse, 5’-TAATGTCACGCACGATTTCC-3’.

### Bioinformatics analysis

First, we predicted lncRNAs targeting miR-130a-3p using StarBase (http://starbase.sysu.edu.cn). Then data from The Cancer Genome Atlas (TCGA) were further used to evaluate the expression levels of the identified top 10 lncRNAs in ccRCC. The most significant up-regulated lncRNA in ccRCC was selected as target lncRNA for the following research. The target lncRNA was further verified in another online database DIANA-LncBase v2[17].

The prognostic value of miR-130a-3p and its interactive lncRNA was analyzed using survival data obtained from TCGA. They were submitted to online analysis tool OncoLnc (http://www.oncolnc.org/) and Kaplan-Meier curve was plotted for analyzing the prognostic value.

### Cell counting kit-8 (CCK-8) assay

CCK-8 (Dojindo, Kumamoto, Japan) assay was applied to explore the proliferation of ccRCC cells. In brief, transfected cells were seeded into 96-well plates at a concentration of 3 × 10^3^ cells/well with 100 uL medium, and continued to be cultured for 24, 48 and 72 h. Then, CCK-8 reagent (10 µL per well) was added to each well at indicated time points. Finally, cell proliferative ability was measured based on the absorbance at 450 nm wavelength by a microplate reader (Thermo Fisher Scientific). The experiment was repeated three times.

### Transwell assay

The invasion of ccRCC cells was evaluated by transwell inserts for 24‑well plates containing 8‑µm pores (Corning Inc., Corning, NY, USA) with a Matrigel-coated membrane (BD Biosciences, San Jose, CA, USA). Cells (1 × 10^5^) in serum‑free DMEM were seeded into the upper chamber of the transwell insert. In the bottom chamber, a total of 800 µl DMEM supplemented with 20% FBS was served as a chemoattractant. After 48 h of incubation in a 37˚C humidified incubator with 5% CO_2_, non-invasive cells on the upper surface were carefully removed with a cotton swab, whereas the invasive cells were fixed with 4% paraformaldehyde for 30 minutes and stained with 0.1% crystal violet for 30 minutes at room temperature. The numbers of invasive cells were counted under a microscope (× 200 magnification) in five random fields. The experiment was performed three times.

### Cell apoptosis measured by flow cytometry

The cell apoptotic ability was measured by flow cytometry using Annexin V-FITC/PI Apoptosis Detection Kit (Wanleibio, Shenyang, China) according to the manufacturer’s protocols. Data collection and analysis were carried out using NovoExpress software (ACEA Biosciences Inc, CA, USA). The apoptotic rates of cells were detected and the exponential values were generated automatically by the software. The apoptosis ratio was calculated as follows: apoptosis ratio (%) = (the percentage of early apoptotic cells) + (the percentage of late apoptotic cells). The percentage of cells is presented in the area of respective quadrant profiles. The assay was repeated three times.

### Dual‐luciferase reporter analysis

According to the binding sites predicted by online bioinformatics tool StarBase, the wild type (WT) and the mutant type (MUT) of CCAT1 were separately amplified by PCR and then cloned into double luciferase reporter vector (pmirGLO Vector). 7.5 nmol miR-130a-3p mimics or miR- negative control, and 1.5 µg luciferase reporters containing CCAT1-WT or CCAT1-MUT were co-transfected into the 293T cells. Using dual-luciferase reporter assay system, we detected the luciferase activity of different groups and confirmed the association between lncRNA CCAT1 and miR-130a-3p. The cells were inoculated into 24-well plates and transfected with miR-130a-3p mimics or the negative control. 48 hours after transfection, dual-luciferase reporting system was used to determine the content. The ratio of firefly luciferase activity to renilla luciferase activity was calculated to evaluate the relative luciferase activity.

### Statistical analysis

We performed statistical analyses using SPSS 23.0 software (Chicago, IL, USA). Graph presentation was carried out using GraphPad Prism software version7.0 (GraphPad Software, La Jolla, CA, USA). The categorical data were expressed as frequencies or percentages, and the continuous data were demonstrated using the mean ± standard deviation (SD). We use Kolmogorov-Smirnov test to analyze the normality. The comparison of rates was performed with χ^2^ test or Fisher exact probability. Using Student’s t-test or one-way ANOVA, the intergroup comparisons of normally distributed continuous variables were performed. Pearson’s correlation coefficient was used to measure the linear correlation. Significant difference was accepted when *P*-value below 0.05.

## Results

### Identification of miRNA-targeted lncRNA

**Fig. 1:**
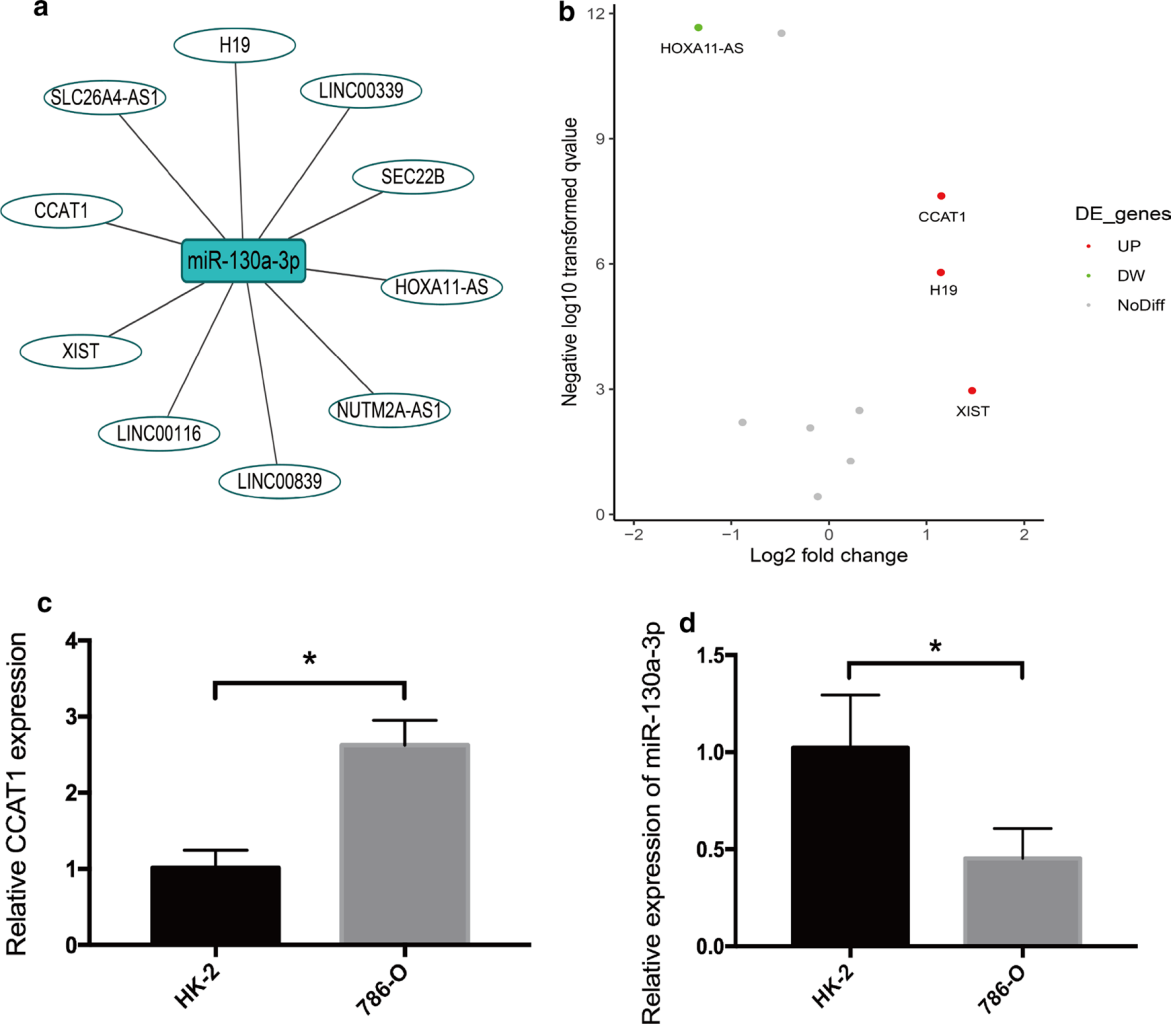
Bioinformatics analysis and mRNA expression of miR-130a-3p and its interactive lncRNAs. **a** The bioinformatics prediction of top ten target lncRNAs of miR-130a-3p by StarBase. **b** CCAT1 is the most significantly upregulated lncRNA in ccRCC by TCGA data. **c** CCAT1 expression is upregulated in ccRCC cell line. *P < 0.05 vs. HK-2 cells. **d** miR-130a-3p expression is downregulated in ccRCC cell line. Comparisons between groups were performed using Student’s t-test. *P < 0.05 vs. HK-2 cells

LncRNAs contain miRNA-response elements, which function as ceRNAs, thereby modulating the derepression of miRNA targets. Therefore, we conducted a bioinformatics analysis to identify the miRNA-targeted lncRNA considering an inverse expression correlation. Using Starbase, we predicted the miRNA-targeted lncRNAs. After screening and matching, we obtained top ten lncRNAs regulated by miR-130a-3p. They are CCAT1, LINC00116, LINC00339, LINC00839, HOXA11-AS, XIST, H19, NUTM2A-AS1, SLC26A4-AS1 and SEC22B (Fig. 1a). Further, we analyzed the differential expression of these lncRNAs in ccRCC and controls by TCGA data, and found that colon cancer associated transcript 1 (CCAT1) was the most significantly up-regulated lncRNA in ccRCC (Fig. 1b). CCAT1 was further verified in another online database DIANA-LncBase v2, which also confirmed the interaction between miR-130a-3p and CCAT1. According to previous research findings, miR-130a-3p may be downregulated and have a tumor-suppressing role in renal carcinoma cells. Therefore, we chose CCAT1 as target lncRNA for subsequent analysis.

### Expression of CCAT1 and miR-130a-3p in cell lines

qRT-PCR was performed to exam the expression of CCAT1 and miR-130a-3p in 786-O and HK-2 cell lines. Our results demonstrated that CCAT1 expression was significantly increased in 786-O cells as compared with HK-2 cells (*P* < 0.05, Fold change = 2.58) (Fig. 1c). Meanwhile, when compared with HK-2 cells, the expression of miR-130a-3p decreased in 786-O cells (*P* < 0.05, Fold change = 0.44) (Fig. 1d).

### **Expression of CCAT1 and miR-130a-3p in human ccRCC tissues**

**Fig. 2:**
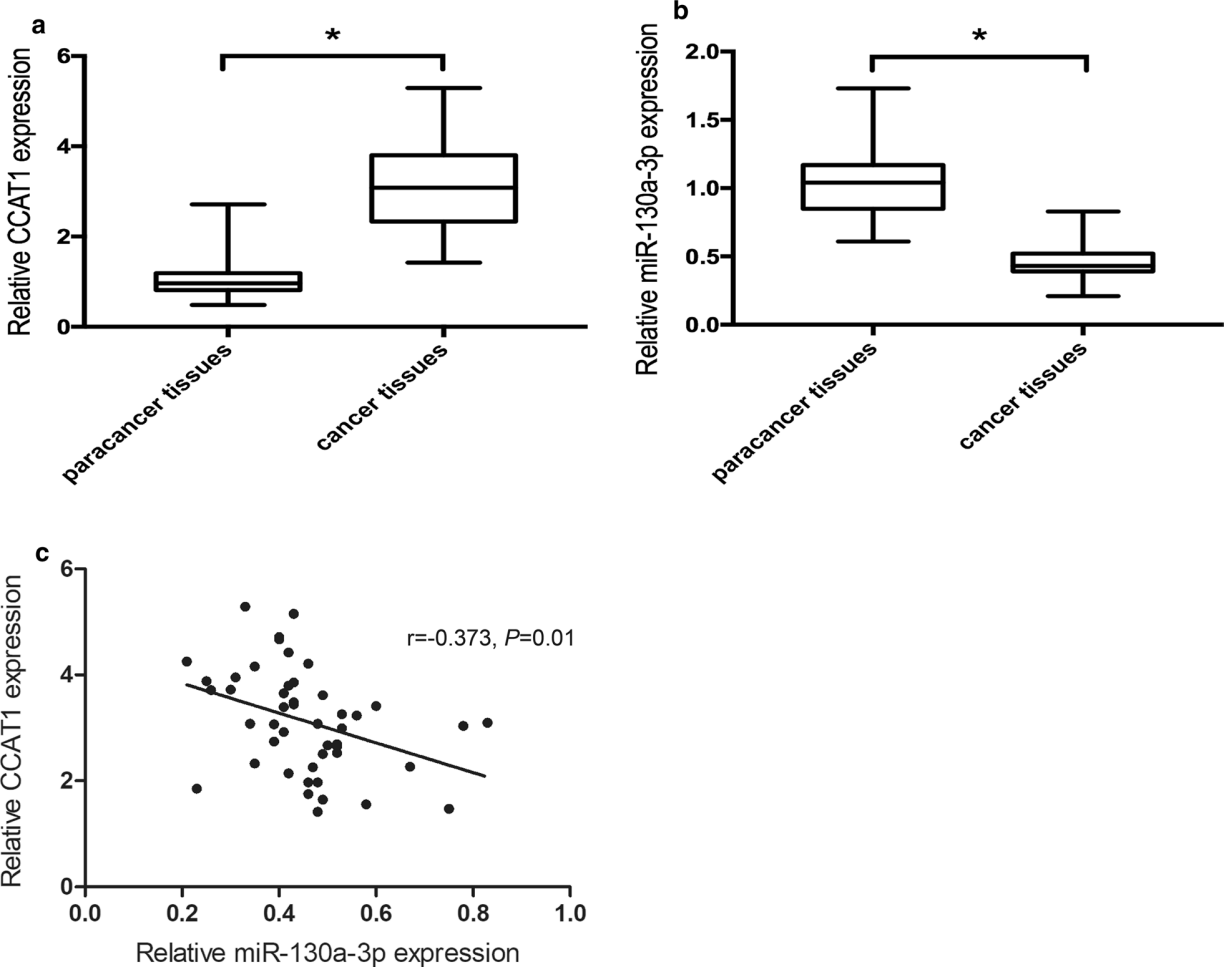
CCAT1 and miR-130a-3p expression in human ccRCC tissues. **a** qRT-PCR analysis of CCAT1 expression. **b** qRT-PCR analysis of miR-130a-3p expression. Comparisons between groups were performed using t-test. *P < 0.05 vs. paracancer tissues. **c** Pearson correlation analysis between between CCAT1 and miR-130a-3p expression in ccRCC tissues

We measured and analyzed the expression of CCAT1 and miR-130a-3p in forty-seven ccRCC patients by qRT-PCR, and found that CCAT1 expression level was significantly increased (*P* < 0.05, Fold change = 2.93), whereas miR-130a-3p level was obviously reduced (*P* < 0.05, Fold change = 0.44) in ccRCC tissues compared with paracancer tissues (Fig. 2a, b). Correlation analysis suggested an inverse relation between CCAT1 and miR-130a-3p expression in ccRCC tissues (r = − 0.373, *P* = 0.010) (Fig. 2c).

Then, we investigated the relationship of CCAT1 and miR-130a-3p expression with the clinicopathological features of ccRCC patients. According to the mean value of CCAT1 and miR-130a-3p expression, ccRCC patients were dichotomized into high and low expression groups. As shown in Table 1, high CCAT1 and low miR-130a-3p expressions were significantly related to bigger tumor size and advanced Tumor Node Metastasis (TNM) stage in ccRCC patients (all *P* < 0.05).

**Table 1:** Association of CCAT1 and miR-130a-3p expression with the clinicopathological characteristics of ccRCC patients

Characteristics	n (%)	CCAT1 expression			miR-130a-3p expression		
		Low, n	High, n	*P* value	Low, n	High, n	*P* value
Gender				0.595			0.676
Male	28(59.6)	14	14		15	13	
Female	19(40.4)	11	8		9	10	
Age, years				0.119			0.642
< 60	27(57.4)	17	10		13	14	
≥ 60	20(42.6)	8	12		11	9	
Location				0.202			0.454
Left	21(44.7)	9	12		12	9	
Right	26(55.3)	16	10		12	14	
Tumor size, cm				*0.014*			*0.011*
≤ 7	28(59.6)	19	9		10	18	
> 7	19(40.4)	6	13		14	5	
Smoking				0.428			0.265
No	36(76.6)	18	18		20	16	
Yes	11(23.4)	7	4		4	7	
BMI, kg/m^2^				0.491			0.871
< 24	21(44.7)	10	11		11	10	
≥ 24	26(55.3)	15	11		13	13	
Thrombus of renal vein				1.000			0.632
No	43(91.5)	23	20		21	22	
Yes	4(8.5)	2	2		3	1	
TNM stage				*0.011*			*0.028*
T_1_-T_2_	34(72.3)	22	12		14	20	
T_3_-T_4_	13(27.7)	3	10		10	3	
ISUP grade				0.560			0.159
1–2	30(63.8)	15	15		13	17	
3–4	17(36.2)	10	7		11	6	

### The prognostic value of CCAT1 and miR-130a-3p in ccRCC

**Fig. 3:**
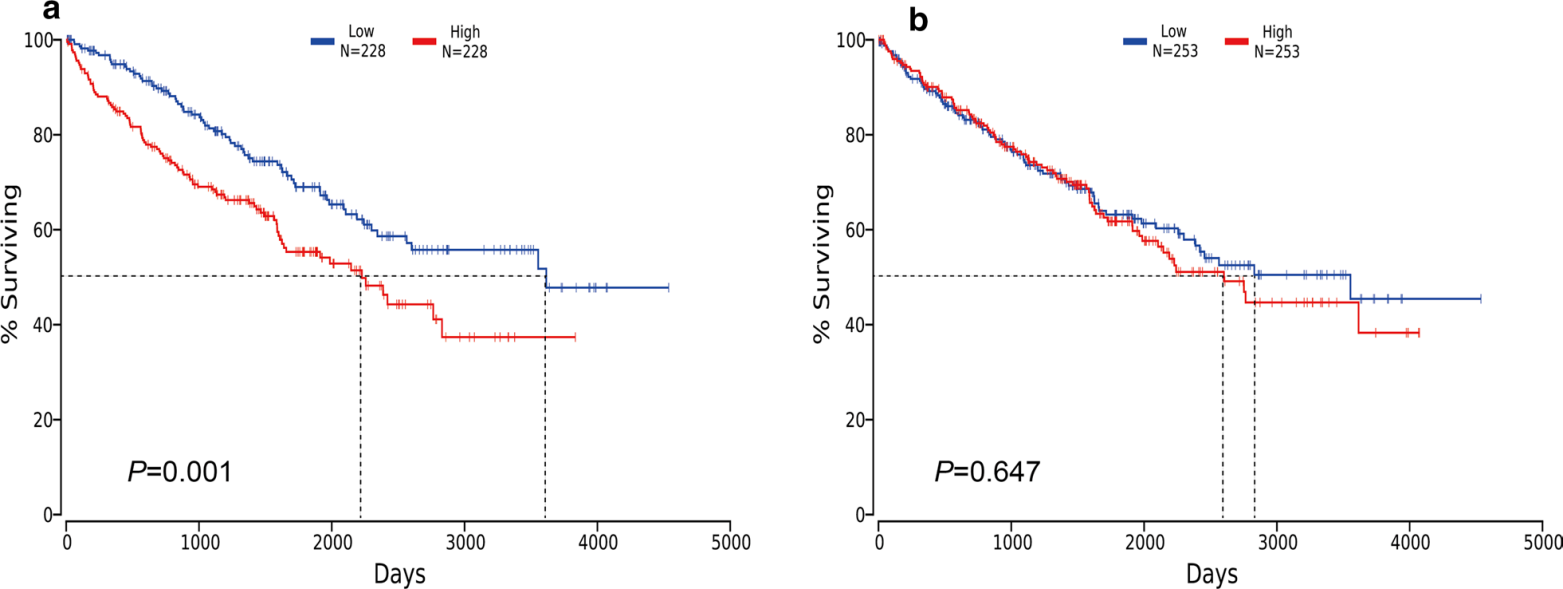
KaplanMeier survival curves are performed to evaluate the prognostic value of **a** CCAT1 and **b** miR-130a-3p in ccRCC

We performed Kaplan-Meier survival curves and log-rank test to evaluate the prognostic value of miR-130a-3p and CCAT1 in ccRCC. The results indicated that the ccRCC patients with high CCAT1 level had a worse survival (*P* = 0.001, Fig. 3a), while there was no significant impact of miR-130a-3p expression on ccRCC prognosis (*P* = 0.647, Fig. 3b).

### MiR-130a-3p inhibits the proliferation and invasion and induces the apoptosis of ccRCC cells

**Fig. 4:**
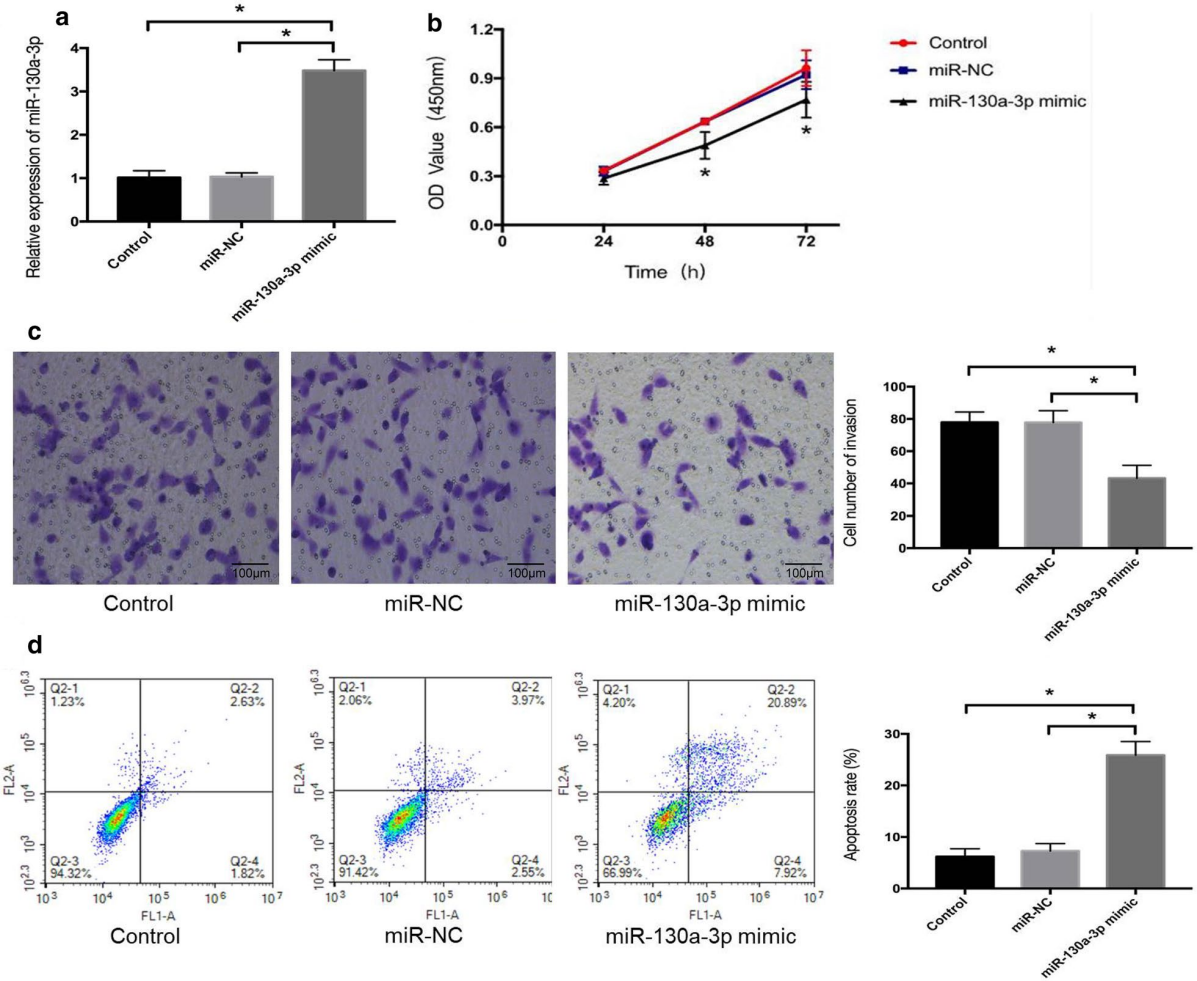
MiR-130a-3p inhibits the proliferation and invasion and induces the apoptosis of ccRCC cells. **a** expression level of miR-130a-3p in different groups. **b** cell proliferation effect was assessed using CCK-8. **c** transwell assay. Representative microscopic images of cells that migrated through the transwell in the migration assay. Scale bar = 100 µm. ×200 magnification. **d **apoptosis effect was assessed using flow cytometry. The apoptosis ratio was calculated as follows: apoptosis ratio (%) = (the percentage of early apoptotic cells) + (the percentage of late apoptotic cells). The percentage of cells is presented in the area of respective quadrant profiles. Comparisons between groups were performed using one-way ANOVA. *P < 0.05

The transfection efficiency of miR-130a-3p mimic in 786-O cells was determined by qRT-PCR. When compared with control and miR-NC group, the expression level of miR-130a-3p was significantly higher in 786-O cells transfected with miR-130a-3p mimic (*P* < 0.05) (Fig. 4a). The results of CCK-8 assay indicated that miR-130a-3p mimic clearly inhibited the proliferation rate of 786-O cells at 48 h and 72 h compared with control and miR-NC group (*P* < 0.05) (Fig. 4b). Transwell assay confirmed that miR-130a-3p mimic significantly suppressed the invasive capacity of 786‑O cells relative to control and miR-NC group (*P* < 0.05) (Fig. 4c). We further investigated the effect of miR-130a-3p on the apoptosis of ccRCC cells. The results of flow cytometry showed that the apoptotic percentage of 786-O cells in control group, miR-NC group and miR-130a-3p mimic group were 6.19 ± 1.52%, 7.28 ± 1.44% and 25.87 ± 2.63% respectively, which suggested that cell apoptosis rate was markedly increased in miR-130a-3p mimic group (*P* < 0.05) (Fig. 4d).

### Knockdown of CCAT1 inhibits the proliferation and invasion and induces the apoptosis of ccRCC cells

**Fig. 5:**
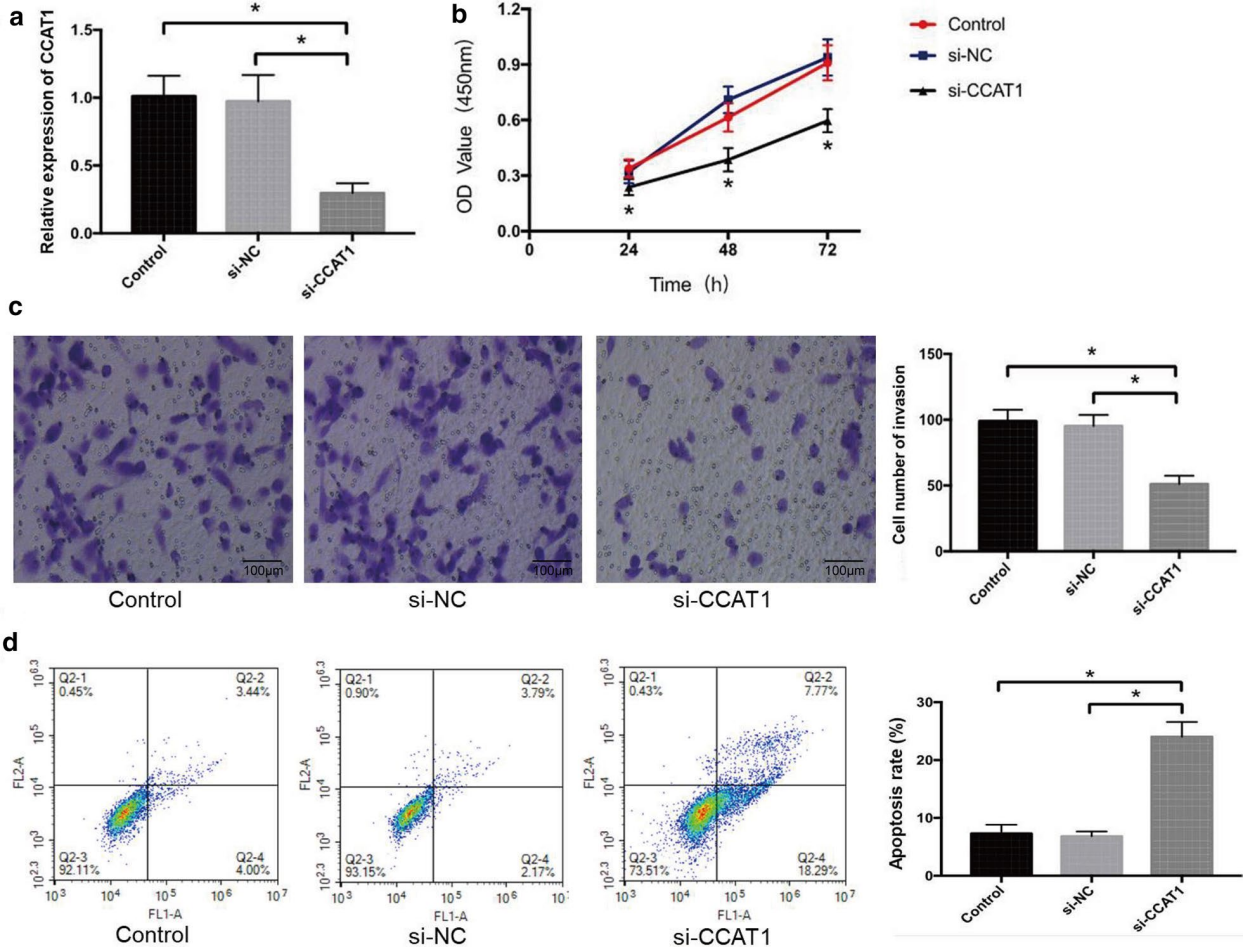
Knockdown of CCAT1 inhibits the proliferation and invasion and induces the apoptosis of ccRCC cells.** a** expression level of miR-130a-3p in different groups. ** b** cell proliferation effect was assessed using CCK-8. ** c** transwell assay. Representative microscopic images of cells that migrated through the transwell in the migration assay. Scale bar = 100 µm. ×200 magnification. ** d** apoptosis effect was assessed using flow cytometry. The apoptosis ratio was calculated as follows: apoptosis ratio (%) = (the percentage of early apoptotic cells) + (the percentage of late apoptotic cells). The percentage of cells is presented in the area of respective quadrant profiles. Comparisons between groups were performed using one-way ANOVA. *P < 0.05

The transfection efficiency of si-CCAT1 in 786-O cells was determined by qRT-PCR. When compared with control and si-NC group, the expression level of CCAT1 was significantly down-regulated in 786-O cells transfected with si-CCAT1 (*P* < 0.05) (Fig. 5a). The results of CCK-8 assay indicated that si-CCAT1 inhibited the proliferation rate of 786-O cells at 48 h and 72 h compared with control and miR-NC group (*P* < 0.05) (Fig. 5b). Transwell assay confirmed that si-CCAT1 significantly suppressed the invasive capacity of 786‑O cells relative to control and si-NC group (*P* < 0.05) (Fig. 5c). We further investigated the effect of CCAT1 on the apoptosis of ccRCC cells. The results of flow cytometry showed that the apoptotic percentage of 786-O cells in control group, si-NC group and si-CCAT1 mimic group were 7.29 ± 1.53%, 6.83 ± 0.84% and 24.03 ± 2.56% respectively, which suggested that cell apoptosis rate was markedly increased in miR-130a-3p mimic group (*P* < 0.05) (Fig. 5d).

### CCAT1 is a ceRNA regulating miR-130a-3p expression in ccRCC cells

**Fig. 6:**
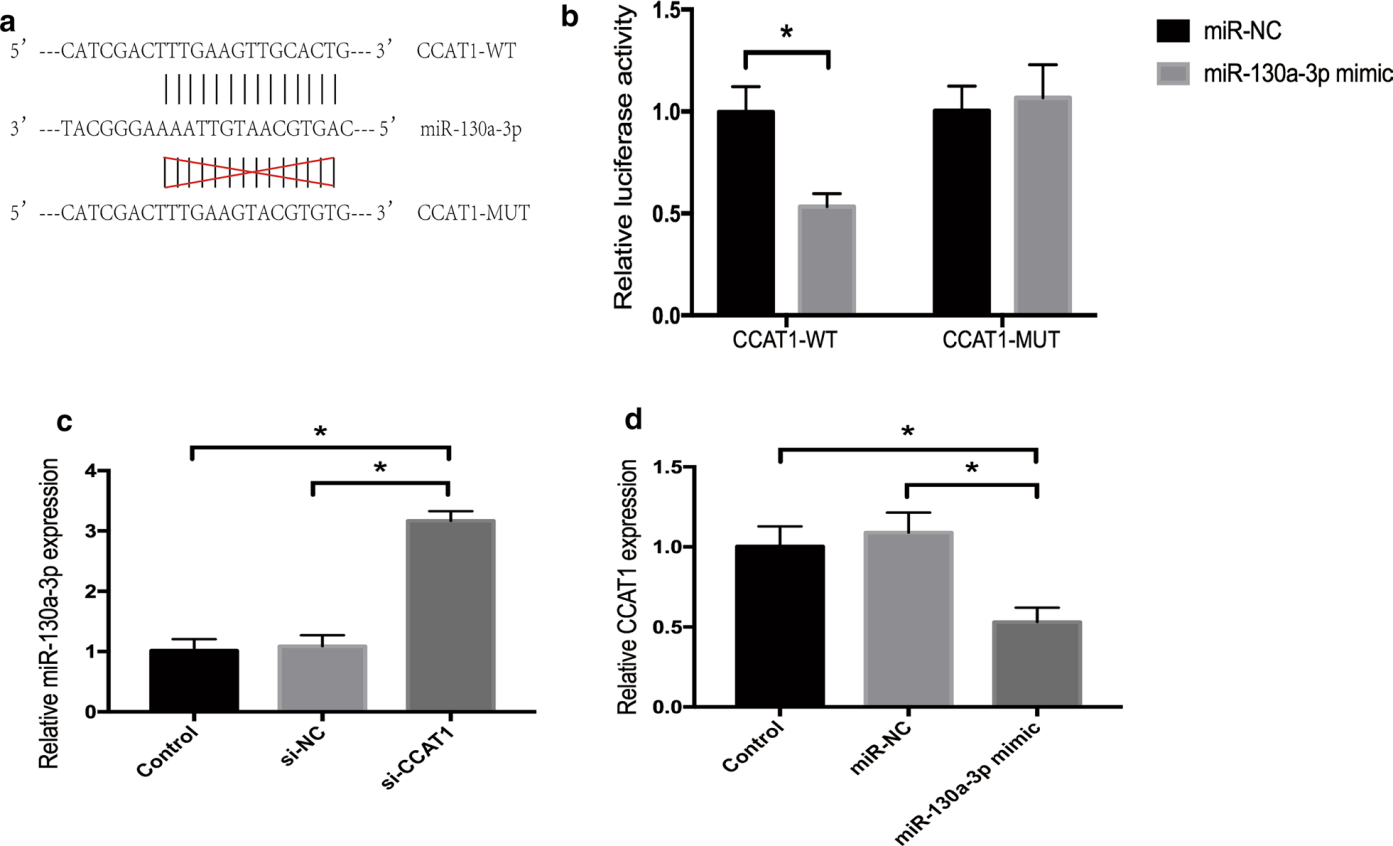
CCAT1 serves as a competing endogenous RNA of miR-130a-3p. **a** The binding sites of miR-130a-3p on CCAT1, as predicted by StarBase. **b** Dual-luciferase reporter assay for direct targeting between CCAT1 and miR-130a-3p. Comparisons between groups were performed using t-test. *P* < 0.05 vs. NC group. **c** miR-130a-3p and **d** CCAT1 expression in 786-O cells after transfection with si-CCAT1 or miR-130a-3p mimic. Comparisons between groups were performed using one-way ANOVA. *P* < 0.05 vs. control or NC group

Using dual-luciferase reporter assay, we further validated whether CCAT1 served as ceRNA of miR-130a-3p. It was found that cotransfection of CCAT1-WT and miR-130a-3p mimics markedly inhibited the luciferase activity in 293T cells (*P* < 0.05), whereas luciferase activity was not obviously changed when cotransfected CCAT1-MUT and miR-130a-3p mimics (Fig. 6a, b).

Furthermore, the regulatory effect of CCAT1 on miR-130a-3p in 786-O cells was measured by qRT-PCR. As shown in Fig. 6c, when compared with control and si-NC groups, miR-130a-3p expression was significantly increased in 786-O cells transfected with si-CCAT1 (*P* < 0.05). In addition, CCAT1 level was reduced in 786-O cells transfected with miR-130a-3p mimics (*P* < 0.05) (Fig. 6d).

## Discussion

For the first time, the current study sought to explore the expression and clinical value of miR-130a-3p and its interactive lncRNA CCAT1 in ccRCC. The results showed that CCAT1 was up-regulated in ccRCC cell line and tissues, and negatively related to miR-130a-3p expression, which significantly decreased in ccRCC cell line and tissues. MiR-130a-3p and knockdown of CCAT1 inhibited the ability of proliferation and invasion and induced the apoptosis of ccRCC cells. Bioinformatics prediction and dual-luciferase reporter analysis validated that CCAT1 owned a putative binding site with miR-130a-3p and had a negative regulation of its expression. Further clinical correlation analyses showed that high CCAT1 and low miR-130a-3p expression had significant effects on aggressive clinicopathological characteristics of ccRCC patients. Moreover, high CCAT1 level was associated with a poor survival prognosis.

ccRCC is a complex disease with multi-step biological procedures, and it is urgent to illuminate the molecular basis of ccRCC in order to understand the molecular mechanism and provide effective therapeutic targets in the future. Accumulating evidence has shown a reciprocal repression between lncRNAs and miRNAs, which can form elaborate networks of regulatory interactions that are associated with plenty of physiological and pathological processes of tumors [18]. miRNAs are being explored as potentially efficient diagnostic and predictive tools as well as therapeutic targets for ccRCC [19], and a recent research has discovered the tumor-suppressing role of miR-130a-3p in renal carcinoma cells[16]. Similarly, in our study, miR-130a-3p also functioned as a tumor suppressor in ccRCC by suppressing cell proliferation and promoting apoptosis. The similar tumor suppressing effect of miR-130a-3p has also been observed in ovarian cancer cells, prostate cancer cells[11, 12], etc. However, some reports showed that miR-130a-3p may serve as an oncogene such as in cervical cancer[15]. Scientific investigations have previously established that miRNAs can either promote or suppress tumor development by mediating different signaling pathways[20]. It is suggested that miRNAs have diverse roles in distinct cell types, and act differently based on their surrounding microenvironment [21]. Therefore, we supposed that miR-130a-3p may have different roles in different circumstances or tissues, and functions differently by targeting different genes in multiple cancers. The potential mechanisms of miR-130a-3p role in different cancers would be an interesting topic.

In this study, we further performed a search for lncRNAs via bioinformatics analysis and identified top ten lncRNAs with tight complementary base pairing with miR-130a-3p. Based on TCGA data, we evaluated the expression levels of selected lncRNAs in ccRCC. Interestingly, CCAT1 was identified as a differentially-expressed lncRNA potentially targeting miR-130a-3p. LncRNA CCAT1, located on chromosome 8q24.21, was initially reported by Nissan et al. in colorectal cancer [22]. As an oncogene, it has attracted more and more attention in the pathogenesis of multiple types of cancers, such as gastric cancer [23], gallbladder cancer [6], esophageal squamous cell carcinoma [24], etc. By using gain- and loss-of-function experiment, CCAT1 was verified to induce proliferation, migration and invasion of tumor cells, and it might also serve as a diagnostic biomarker in some relevant cancers [25]. The biological function of CCAT1 in renal cell carcinoma has been preliminarily investigated by Chen et al. They observed that CCAT1 expression was increased in renal cell carcinoma tissues as well as cell lines, and inhibited the apoptosis and enhanced the viability of renal cell carcinoma cells [26]. In our study, knockdown of CCAT1 inhibited the proliferation and invasion and induced the apoptosis of ccRCC cells, which suggested the tumor-promoting function of CCAT1 in ccRCC.

However, it is still unknown about the regulatory relationship between CCAT1 and miR-130a-3p in ccRCC. In our study, the up-regulation of CCAT1 and down-regulation of miR-130a-3p were observed in ccRCC cell line, and relative luciferase activity decreased in cells co-transfected with CCAT1-WT and miR-130a-3p mimics, indicating the binding of miR-130a-3p with the sequence of CCAT1. Then, in 786-O model, it was observed that CCAT1 could negatively regulate the expression of miR-130a-3p by directly interacting with miR-130a-3p. In detail, CCAT1 silencing significantly increased miR-130a-3p expression, while miR-130a-3p mimics remarkably suppressed CCAT1 expression. The biological roles of miR-130a-3p and CCAT1 in regulating ccRCC progression were also investigated *in vitro* in our experiments, which suggested that miR-130a-3p and knockdown of CCAT1 inhibited ccRCC cell proliferation and invasion, and induced apoptosis. According to these results, we speculated that CCAT1 could serve as a molecular sponge in promoting tumor progression by downregulating miR-130a-3p in ccRCC, and there was a reciprocal interaction between CCAT1 and miR-130a-3p. Previous literatures have well documented that lncRNA CCAT1 could negatively regulate miRNAs. In addition, due to the similarity between lncRNA and mRNA, miRNA may negatively regulate the expression of lncRNA through a mechanism similar to mRNA[27]. Until now, only one study has discussed the contribution of CCAT1/miR-130a-3p axis to the cell biological functions. Hu et al. found that CCAT1 increased while miR-130a-3p decreased in cisplatin-resistant non-small-cell lung cancer (NSCLC) cells, and CCAT1 participated in cisplatin resistance of NSCLC cells by downregulating miR-130a-3p [28]. The in-depth mechanism of this reciprocal interaction between CCAT1 and miR-130a-3p in ccRCC was required to be elucidated in future studies.

CCAT1 has been reported to be consistently up-regulated in a variety of cancer tissues and participate in the occurrence and metastasis of various cancers [18, 29]. The overexpression of CCAT1 was closely related to aggressive clinical parameters in relevant cancers, including tumor size, TNM stage, lymph node metastasis and vascular invasion [25, 30–32]. The down-regulation of miR-130a-3p was reported in human nasopharyngeal carcinoma and breast cancer tissues, and low expression of miR-130a-3p was closely associated with lymph node metastasis and TNM stage of breast cancer [33, 34]. Further, we conducted a comprehensive investigation on the clinical relevance of CCAT1 and miR-130a-3p in ccRCC patients. The results showed that CCAT1 level was increased in ccRCC tissues, which was consistent with a previous study [26], while miR-130a-3p expression was aberrantly decreased in ccRCC specimens relative to paracancer tissues. Moreover, correlation analysis demonstrated that CCAT1 and miR-130a-3p expressions were negatively correlated in ccRCC tissues, indicating a functional interaction between CCAT1 and miR-130a-3p in ccRCC. Additionally, the relationship of CCAT1 and miR-130a-3p with clinicopathological parameters of ccRCC was analyzed. We found that high CCAT1 and low miR-130a-3p expression levels were related to larger tumor size and advanced TNM stage in ccRCC patients, indicating the possible role of CCAT1 and miR-130a-3p as progression monitoring indicators and therapeutic targets in ccRCC.

Meanwhile, it is of great clinical value to seek for prognostic biomarkers of ccRCC, which can sensitively predict therapeutic outcomes and help to propose appropriate treatment options. Studies have confirmed the relationship between CCAT1 and poor prognosis in several cancers [35–37]. Notably, CCAT1 was inversely related to the overall survival as well as progression-free survival in breast cancer [38], while higher CCAT1 level was associated with shorter survival time in ovarian cancer [31]. Based on two quantitative meta-analyses, CCAT1 was proved to serve as an independent prognosis factor to evaluate clinical outcomes of tumor patients [25, 32]. The only research about the role of miR-130a-3p in predicting cancer prognosis observed that the overexpressed miR-130a-3p was correlated with a worse outcome of cardiac cancer patients, and appeared to alter the radio- and chemo-sensitivities of cardiac cancer [39]. However, to date, it is unclear about the prognostic significance of CCAT1 and miR-130a-3p in ccRCC. In this study, we obtained survival data from TCGA to further investigate the prognostic role of CCAT1 and miR-130a-3p in ccRCC. Kaplan-Meier analysis showed that high expression of CCAT1 had a significant impact on a poor survival prognosis, indicating that CCAT1 may act as a prognostic biomarker for ccRCC. However, our results failed to demonstrate an association between miR-130a-3p and ccRCC prognosis. MiR-130a-3p may have different roles in different circumstances or stages of cancer. It may play a role mainly in the tumorigenesis stage instead of prognosis of ccRCC. In addition, the current results were based on the bioinformatics analysis. In the future, we should collect prognostic information and further verify the exact relationship between mir-130a-3p and prognosis in the population.

In the current study, a few limitations should be underlined. Our prognostic results were based on bioinformatics and lack of clinical validation. The size and constitution of the included samples were limited, which could not be stratified for further analyses, and a larger ccRCC cohort needed to be gathered. The detailed mechanism requires further exploration through RIP/RNA pull down, gain- and loss-of-function experiments *in vitro* and *vivo* in the future.

## Conclusions

Taken together, bioinformatics analysis along with biological experiments indicated that CCAT1 might function as a molecular sponge of miR-130a-3p to participate in the pathogenesis of ccRCC. MiR-130a-3p and knockdown of CCAT1 inhibited ccRCC cell proliferation and invasion, and induced apoptosis. High CCAT1 and low miR-130a-3p levels were revealed in ccRCC cell line and tissues. Moreover, CCAT1 and miR-130a-3p expression in ccRCC tissues was related to tumor size and TNM stage, and CCAT1 was associated with the prognosis of ccRCC. Therefore, CCAT1/miR-130a-3p axis may play a potential role in assessing ccRCC diagnosis and prognosis. Further studies of the molecular mechanisms underlying the role of CCAT1/miR-130a-3p axis in the occurrence and progression of ccRCC are warranted.

## Data Availability

The authors declare that the data supporting the findings of this study are available within the article.

## Abbreviations

ncRNAs: Noncoding RNAs
miRNAs: microRNAs
lncRNAs: Long non-coding RNAs
ceRNAs: Competing endogenous RNAs
ccRCC: Clear cell renal cell carcinoma
CCAT1: Colon cancer associated transcript 1
TCGA: The Cancer Genome Atlas
CCK-8: Cell counting kit-8
WT: Wild type
MUT: Mutant type
SD: Standard deviation
TNM: Tumor Node Metastasis
NSCLC: Non-small-cell lung cancer
